# Heart failure with preserved ejection fraction is the most frequent but commonly overlooked phenotype in patients on chronic hemodialysis

**DOI:** 10.3389/fcvm.2023.1130618

**Published:** 2023-06-01

**Authors:** Jan Malik, Anna Valerianova, Satu Sinikka Pesickova, Kristyna Michalickova, Zuzana Hladinova, Zdenka Hruskova, Vladimira Bednarova, Katarina Rocinova, Monika Tothova, Marketa Kratochvilova, Lucie Kaiserova, Kristina Buryskova Salajova, Vaclav Lejsek, Martin Sevcik, Vladimir Tesar

**Affiliations:** ^1^ 3rd Department of Internal Medicine, First Faculty of Medicine, General University Hospital, Charles University, Prague, Czechia; ^2^ Dialysis Center Ohradni, B. Braun Avitum, Prague, Czechia; ^3^ Dialysis Center Taborska, B. Braun Avitum, Prague, Czechia; ^4^ Department of Nephrology, General University Hospital, First Faculty of Medicine, Charles University, Prague, Czechia; ^5^ Dialysis Center Cerny Most, B. Braun Avitum, Prague, Czechia; ^6^ Dialysis Center Motol, Fresenius Medical Care, Prague, Czechia; ^7^ Dialysis Center Uhersky Brod, B. Braun Avitum, Uhersky Brod, Czechia

**Keywords:** heart failure, end-stage renal disease, HFPEF, high-output heart failure, echocardiography

## Abstract

**Introduction:**

Heart failure (HF) is a serious complication of end-stage kidney disease (ESKD). However, most data come from retrospective studies that included patients on chronic hemodialysis at the time of its initiation. These patients are frequently overhydrated, which significantly influences the echocardiogram findings. The primary aim of this study was to analyze the prevalence of heart failure and its phenotypes. The secondary aims were (1) to describe the potential of N-terminal pro-brain natriuretic peptide (NTproBNP) for HF diagnosis in ESKD patients on hemodialysis, (2) to analyze the frequency of abnormal left ventricular geometry, and (3) to describe the differences between various HF phenotypes in this population.

**Methods:**

We included all patients on chronic hemodialysis for at least 3 months from five hemodialysis units who were willing to participate, had no living kidney transplant donor, and had a life expectancy longer than 6 months at the time of inclusion. Detailed echocardiography together with hemodynamic calculations, dialysis arteriovenous fistula flow volume calculation, and basic lab analysis were performed in conditions of clinical stability. Excess of severe overhydration was excluded by clinical examination and by employing bioimpedance.

**Results:**

A total of 214 patients aged 66.4 ± 14.6 years were included. HF was diagnosed in 57% of them. Among patients with HF, HF with preserved ejection fraction (HFpEF) was, by far, the most common phenotype and occurred in 35%, while HF with reduced ejection fraction (HFrEF) occurred only in 7%, HF with mildly reduced ejection fraction (HFmrEF) in 7%, and high-output HF in 9%. Patients with HFpEF differed from patients with no HF significantly in the following: they were older (62 ± 14 vs. 70 ± 14, *p* = 0.002) and had a higher left ventricular mass index [96(36) vs. 108(45), *p* = 0.015], higher left atrial index [33(12) vs. 44(16), *p* < 0.0001], and higher estimated central venous pressure [5(4) vs. 6(8), *p* = 0.004] and pulmonary artery systolic pressure [31(9) vs. 40(23), *p* = 0.006] but slightly lower tricuspid annular plane systolic excursion (TAPSE): 22 ± 5 vs. 24 ± 5, *p* = 0.04. NTproBNP had low sensitivity and specificity for diagnosing HF or HFpEF: with the use of the cutoff value of 8,296 ng/L, the sensitivity of HF diagnosis was only 52% while the specificity was 79%. However, NTproBNP levels were significantly related to echocardiographic variables, most significantly to the indexed left atrial volume (*R* = 0.56, *p* < 10^−5^) and to the estimated systolic pulmonary arterial pressure (*R* = 0.50, *p* < 10^−5^).

**Conclusions:**

HFpEF was by far the most common heart failure phenotype in patients on chronic hemodialysis and was followed by high-output HF. Patients suffering from HFpEF were older and had not only typical echocardiographic changes but also higher hydration that mirrored increased filling pressures of both ventricles than in those of patients without HF.

## 1. Introduction

Heart failure (HF) with preserved ejection fraction (HFpEF) is a clinical syndrome characterized by HF symptoms, left ventricular ejection fraction (LVEF) ≥ 50%, and evidence of cardiac dysfunction (e.g., abnormal LV filling and elevated filling pressures) (1, 2). In the absence of pericardial or valvular disease, diastolic left ventricular dysfunction results from increased stiffness (of cardiomyocytes and extracellular matrix) and/or slower relaxation (1). The arterial wall also changes due to decreased proportion of elastin and calcium deposition. Thus impaired ventriculoatrial coupling also characterizes HFpEF (3). These changes are accelerated in patients with chronic kidney disease.

The prevalence of chronic kidney disease (CKD) is growing worldwide. In developed countries, this is mainly due to the epidemics of obesity and metabolic syndrome that include two important causes of CKD—hypertension and type 2 diabetes mellitus. Patients, whose CKD progresses into end-stage kidney disease (ESKD), need renal function replacement therapy. Hemodialysis is the most frequent method by far. However, ESKD and hemodialysis itself are associated with vast hemodynamic, metabolic, and endocrine changes that lead to significant functional and morphological changes in many organs, including the cardiovascular system (4). Indeed, cardiovascular complications are responsible for significant morbidity and represent the most frequent cause of death in this population. Moreover, cardiovascular changes develop much faster in ESKD patients than that in the general population, and this acceleration of pathological changes is responsible for the shorter lifespan of ESKD patients (5). Practically, all heart structures could be affected (4), and heart failure (HF) is a frequent consequence.

CKD and especially ESKD are characterized by sodium and water retention and thus with a high risk of overhydration. In patients on chronic hemodialysis, fluids are removed by ultrafiltration, which is directed by the difference between actual and dry weight (weight of the patient at the end of dialysis sessions). Setting an appropriate dry weight is difficult and based on clinical experience, lab results, and bioimpedance. Therefore, water overload is not rare in hemodialysis patients. Its signs and symptoms are very similar to heart failure (6) and chronic overload is associated with higher mortality (7). Distinguishing water overload from real HF is therefore not easy, especially for retrospective studies analyzing hospital discharge letters. Interestingly, most of our knowledge about HF prevalence in ESKD patients on hemodialysis comes from such retrospective studies (8, 9).

Briefly, HF seems to be frequent among patients on chronic hemodialysis, and many mechanisms have been discovered. However, most data come from retrospective studies that did not quantify the role of actual hydration and where the diagnosis of HFpEF was not based on the current recommendations. To overcome these limitations, we started a prospective observational study named CZecking HF in CKD (10) (registered as ISRCTN 18275480) in 2020. Here we present the baseline data with regard to HF prevalence and phenotypes in a central European population. All patients were examined by detailed expert echocardiography, and lack of significant overhydration was confirmed by both ultrasonography and bioimpedance. The primary aim of this study was to analyze the prevalence of heart failure and its phenotypes. The secondary aims were (1) to describe the potential of N-terminal pro-brain natriuretic peptide (NTproBNP) for HF diagnosis in ESKD patients on hemodialysis; (2) to analyze the frequency of abnormal left ventricular geometry; and (3) to describe the differences between various HF phenotypes in this population.

## 2. Materials and methods

In the CZecking HF in CKD study, all prevalent patients on chronic hemodialysis (>3 months) that fulfill the broad eligibility criteria came for the index examination, after which they were followed every 6–12 months lifelong or till kidney transplantation. The study design has already been published (10), and the inclusion of patients is ongoing. Briefly, all patients of collaborating hemodialysis units were asked to participate in this study, and the exclusion criteria were only planned kidney transplantation from a living donor within 3 months of the index visit or life expectancy <6 months at V1 visit of any reason. Here, we present baseline data (Visit 1) of patients included till 30 June 2022. The study was approved by the Ethical Committee of the General University Hospital in Prague and is registered in the ISRCTN database. We explained the principles of the study to all patients, and they signed informed consent.

The following data were recorded: basic medical history data, full blood count, levels of albumin, total blood protein and NTproBNP, echocardiography, volume status estimation, heart rhythm analysis, and hemodialysis fistula flow volume calculation. Pulmonary arterial systolic pressure was estimated as a sum of the central venous pressure and the tricuspid regurgitation gradient (if present).

Expert echocardiography was performed using a matrix echocardiography probe of Vivid E9 device (General Electric, Vingmed, Norway), as well as detailed analysis of the volumes of heart cavities, quantification of valvular disease, diastolic dysfunction according to the recent guidelines (11), and cardiac output calculation (using the left ventricular outflow tract diameter and velocity time interval). Echocardiography and all other examinations were performed within 1 h on a non-dialysis day. Two examiners experienced in cardio-nephrology provided all examinations (AV, JM).

Hydration status was assessed as the central venous pressure with the use of the diameter and collapsibility of the inferior vena cava (12) and by bioimpedance (Body Composition Monitor (BCM), Fresenius Medical Care (FMC), Germany). Patients with significant overhydration (>5 L) were not included. Hemodialysis fistula flow volume was analyzed by duplex Doppler ultrasonography at the level of the brachial artery as described previously (13). Hemodynamic calculations (effective cardiac output, vascular resistance, access resistance) were based on echocardiographic data and vascular access flow measurement.

Heart failure diagnosis is established by the guidelines of the European Society of Cardiology (14). HF phenotypes were diagnosed according to the same guidelines (HFpEF), HF with mildly reduced ejection fraction (HFmrEF), HF with reduced ejection fraction (HFrEF)), and two other phenotypes were added: (1) high-output heart failure (HOHF defined by HF signs and symptoms + cardiac index >3.9 L/min/m^2^) and (2) significant primary valvular disease. The diagnosis of heart failure was set by a cardiologist experienced in cardio-nephrology (JM, AV) and was based on a combination of HF signs and symptoms that improve after each hemodialysis and documentation of structural heart disease by echocardiography. The latter was based on the modified ADQI criteria (10) (see Table 1).

**Table 1:** Modified ADQI echocardiographic criteria of heart failure.

Major criterion	Minor criteria	Pathological values
EF < 40%	Diastolic dysfunction	Stage 2 or 3 or atrial fibrillation + left atrial dilatation according to (15)
Significant valvular disease (16)	Left ventricular hypertrophy	Indexed left ventricular mass >95 g/m^2^ in women and >115 g/m^2^ in men
	Left ventricular dilatation	End-diastolic volume >61 ml/m^2^ in women and >74 ml/m^2^ in men
	Pulmonary hypertension	Estimated systolic right ventricular pressure >35 mmHg in subjects with tricuspid regurgitation
	Coronary artery disease	RWMA + ≥1 stenosis > 50% or history of intervention OR history of myocardial infarction + stable RWMA
	EF 40%–50%	
	Right ventricular dysfunction	TAPSE < 18 mm
	Moderate valvular disease	Stenosis or stable regurgitation according to (16)

EF, ejection fraction (left ventricular); RWMA, regional wall motion abnormality; TAPSE, tricuspid annular plane systolic excursion.

Assessments were performed according to the official guidelines as stated by the references. The ejection fraction was calculated by the biplane methods of disks as recommended (11). For the HF diagnosis according to this definition, the presence of at least one major or two minor criteria was necessary. We also recorded the diagnosis of any HF phenotype according to the nephrologists (based on the clinical picture, patients’ history, and/or echocardiography performed elsewhere in the history).

### 2.1. Statistical analysis

The normality of data distribution was tested by the Lilliefors test. Data are reported as mean ± SD in variables with Gaussian distribution or as median (quartile range) in variables with non-Gaussian distribution. The difference between (sub) groups was tested by unpaired *t*-test in the case of Gaussian distribution of data and by Mann­–Whitney *U* test. The relation between variables was calculated by the Spearman correlation analysis. Receiver-operating characteristics were calculated to be considered predictors of HF or, specifically, HFpEF.

## 3. Results

We report data from 214 patients, aged 66.4 ± 14.6 years, of which 65% were males, the mean dialysis vintage is 47 months (median 26 months), and 98.5% of included patients had Caucasian race. The most frequent causes of CKD were diabetes mellitus (31%), hypertension (23%), and polycystic kidney disease (7%). Hemodialysis vascular access was arteriovenous fistula in 73%, arteriovenous graft in 19%, and catheter in 8%. Other baseline data are presented in Table 2. Eight selected patients were not included in this analysis because of significant overhydration as defined above. A decrease in the dry weight setting was recommended, and the patients were re-examined and then entered the main study.

**Table 2:** Baseline characteristics of all included patients.

Heart rate (min^−1^)	73 (15)
Systolic blood pressure (mmHg)	132 ± 27
Diastolic blood pressure (mmHg)	73 ± 16
Mean arterial pressure (mmHg)	112 ± 22
Body mass index (kg/m^2^)	26 (7)
Shortness of breath—NYHA	2 (1)
Residual diuresis (ml/24 h)	500 (1,000)
NTproBNP (ng/L)	4,772 (11,988)
Hemoglobin (g/L)	110 (18)
Total protein (g/L)	65 (7)
Albumin (g/L)	38 (4)
Left ventricular mass index (g/m^2^)	106 (42)
Cardiac index (L/min/m^2^)	3.19 (1.01)
Ejection fraction (%)	57.8 (11.9)
Left atrial volume indexed (ml/m^2^)	40 (21)
Right atrial volume (ml)	64 (52)
Central venous pressure (mmHg)	5 (6)
Right ventricular systolic pressure (mmHg)	35 (21)
Dialysis access flow volume (ml/min)	1,041 (776)

Data are reported as mean ± SD in variables with Gaussian distribution or as median (quartile range) in variables with non-Gaussian distribution.

NYHA, New York Heart Association; NTproBNP, N-terminal pro-brain natriuretic peptide.

**Figure 1:**
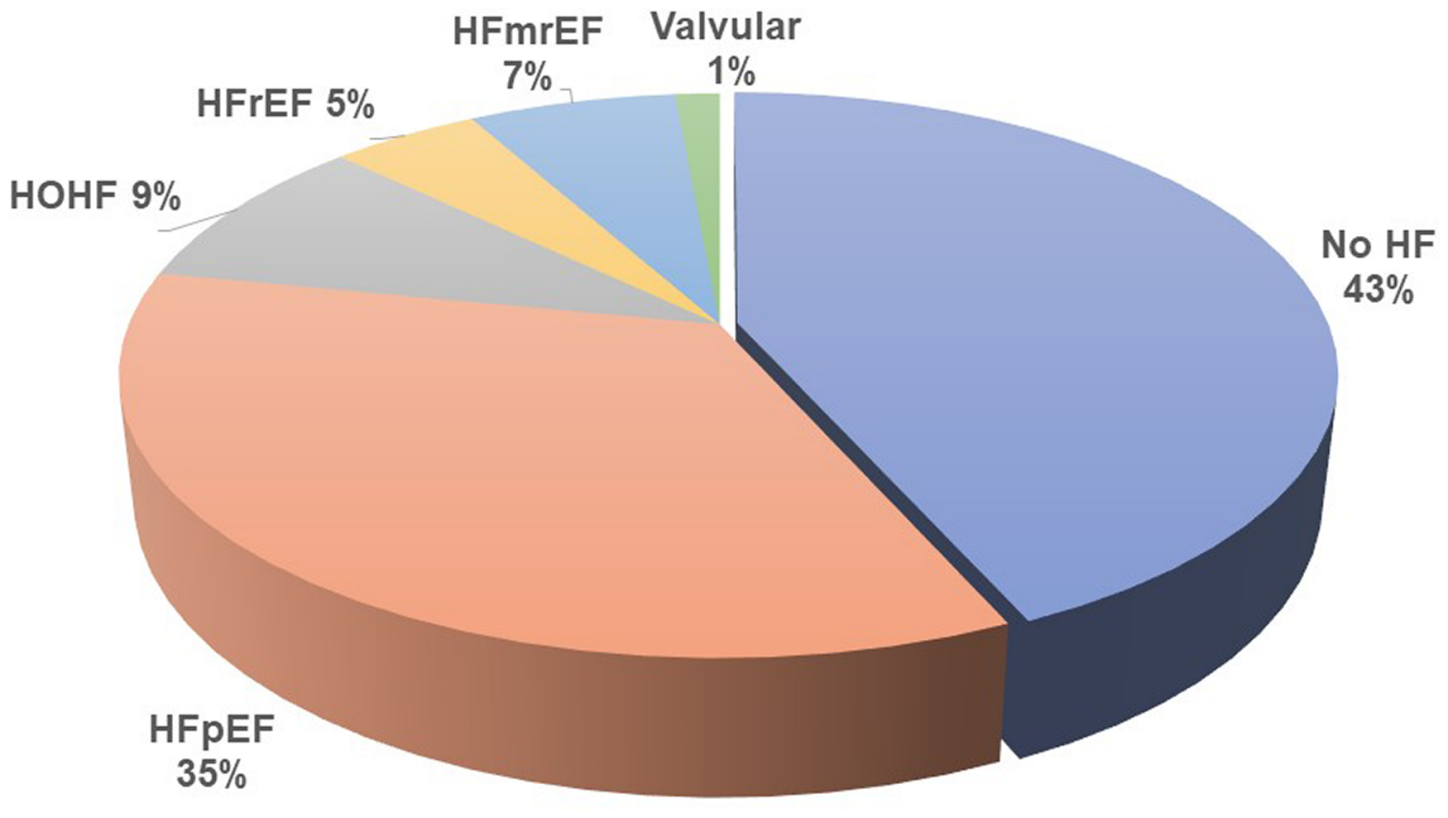
Heart failure and its phenotypes distribution in patients on chronic hemodialysis.

Heart failure was diagnosed in 122 (57%) of all included patients by the cardiologists, but only in 25% by the referring nephrologists. HFpEF was the leading HF phenotype (see Figure 1). Patients suffering from HFpEF were older and had higher left ventricular mass, left atrial volume, and pulmonary artery blood pressure (see Table 3 for details). The referring nephrologists diagnosed correctly significant valvular disease in 100%, HFrEF in 80%, HFmrEF in 43%, HFpEF in 31%, and HOHF in 18%.

**Table 3:** Comparison of heart failure phenotypes.

Variable	No HF	HFpEF	HFrEF	HFmrEF	HOHF
Number of cases	92	75	11	14	19
Age (years)	65.5 (20)	74 (16)***	76 (16)*	70 (18)	66 (21)
Dialysis vintage (months)	24 (54)	24 (54)	77 (109)	38 (39)	38 (99)
Body mass index kg/m^2^	25.7 (7.5)	26.9 (9.0)*	24.2 (8.4)	26.3 (7.6)	25.2 (7.6)
Systolic blood pressure (mmHg)	127 ± 27	139 ± 27**	123 ± 20	120 ± 20	144 ± 24*
Diastolic blood pressure (mmHg)	73 ± 16	75 ± 15	66 ± 9	61 ± 13**	80 ± 17
Mean blood pressure (mmHg)	109 ± 22	118 ± 21*	108 (16)	100 ± 17	123 ± 20*
Heart rate (1/min)	74 (15)	74 (14)	71 (24)	68 (16)	79 (14)
Shortness of breath (NYHA)	1.0 (1.0)	2.0 (1.0)***	3.0 (2.0)**	3.0 (2.0)***	2.0 (1.0)**
LV mass index (g/m^2^)	96 (36)	108 (45)*	138 (59)**	123 (28)***	115 (25)**
LV ejection fraction (%)	65 (20)	59 (12)	34 (16)	44 (6)	61 (14)
Cardiac index (L/min/m^2^)	3.17 (0.95)	3.15 (0.85)	2.76 (0.90)*	2.79 (1.55)	4.64 (1.3)***
Left atrial volume index (ml/m^2^)	33 (17)	44 (16)***	62 (15)***	48 (15)**	47 (22)***
TAPSE (mm)	24 (6)	23 (6)	16 (4)**	21 (4)	25 (5)*
Central venous pressure (mmHg)	5 (4)	6 (8)**	14 (10)***	5 (5)*	8 (7)***
RV systolic pressure (mmHg)	31 (9)	40 (23)**	52 (20)**	43 (15)	37 (22)
Qa (ml/min)	950 (800)	880 (760)	750 (1,100)	968 (625)	1,900 (2,100)***
Effective cardiac index (L/min/m^2^)	2.53 (0.92)	2.58 (0.95)	2.24 (1.04)	2.03 (0.63)	3.74 (0.78)***
Qa/CO (%)	18.7 (14.3)	16.3 (10.9)	16.4 (21.4)	18.2 (14.7)	16.3 (17.7)
TVR (Wood units)	17.5 (7.5)	19.7 (7.8)	17.7 (10.8)	18.9 (13.9)	12.1 (3.4)***
SVR (Wood units)	22.2 (7.1)	22.9 (10.6)	20.7 (8.8)	25.8 (17.6)	14.8 (2.9)***
AVF resistance (Wood units)	91 (59)	127 (66)*	58 (42)	109 (81)	83 (69)
Diastolic (dys)function (grade)	0 (2)	2 (1)***	2 (1)***	2 (1.5)	2 (0)***
NTproBNP (ng/L)	3,011 (7,425)	2,781 (3,090)***	28,010 (21,960)**	6,733 (13,774)*	2,781 (3,090)
Hemoglobin (g/L)	111 (16)	109 (17)	112 (24)	108 (17)	103 (16)

LV, left ventricle; TAPSE, tricuspid annular plane systolic excursion; RV, right ventricle; Qa, dialysis access flow volume. AVF, arteriovenous fistula, CO, cardiac output; TVR, total vascular resistance (including Qa); SVR, systemic vascular resistance.

* *p* < 0.05.

** *p* < 0.01.

*** *p* < 0.001 versus no heart failure. This table does not include patients with primary valvular disease.

### 3.1. Other findings

Normal left ventricular geometry was present in only 20% of patients. Out of the patients with abnormal geometry, 44% had concentric hypertrophy, 33% had concentric remodeling, and 24% had eccentric hypertrophy. The left ventricular diastolic function could be formally assessed in 158 (74%) patients (see Table 4).

**Table 4:** Left ventricular diastolic function.

Diastolic (dys)function	Cases (% of all assessable)
Normal diastolic function	50 (30%)
Grade 1 dysfunction	28 (17%)
Grade 2 dysfunction	73 (43%)
Grade 3 dysfunction	7 (4%)

Left ventricular dilatation was diagnosed in 24% of all patients. Pulmonary artery systolic blood pressure estimation was possible in 122 patients, and, in those, pulmonary hypertension (defined by the estimated peak systolic pulmonary pressure of 35 mmHg or higher) occurred in 56 (46%) patients. The non-sinus rhythm was recorded in 19% of examinations.

**Figure 2:**
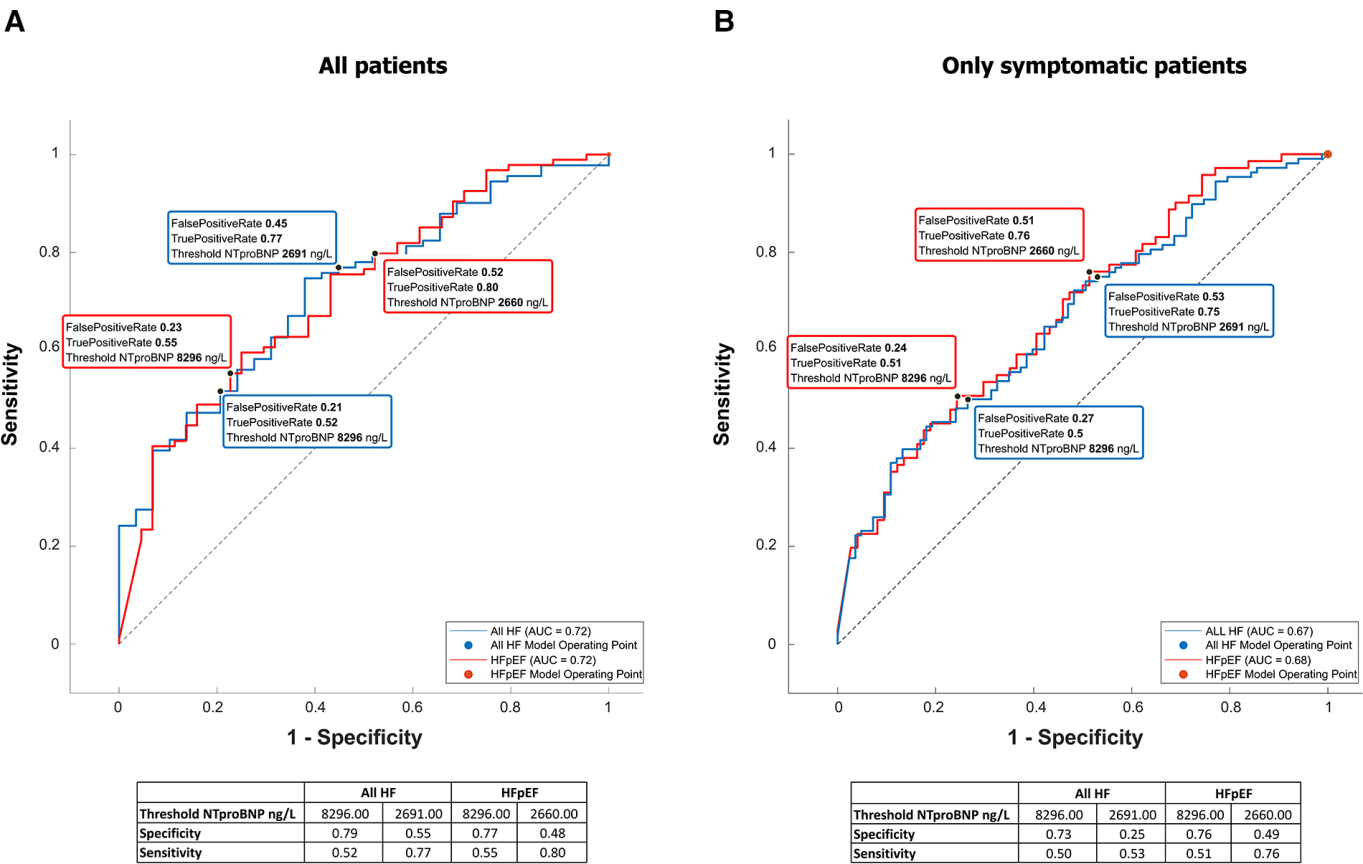
NTproBNP for the diagnosis of heart failure and HFpEF. Receiver-operating curves for NTproBNP differentiating between HF or specifically HFpEF and no HF. The left graph (**A**) is based on all included patients, while the right one (**B**) included only patients with shortness of breath (NYHA II–IV).

NTproBNP was high in the whole group (see Table 1 for details) but significantly higher in patients with any type of HF [6,935 (17,575) vs. 3,011 (7,425), *p* = 0.00007]. It was significantly related to many variables tested in this study (Table 5), and by most, it was positively related to the left atrial volume and the estimated peak pulmonary systolic pressure. However, NTproBNP was inversely related to the left ventricular ejection fraction in this study. This biomarker had low sensitivity and specificity for diagnosing HF or HFpEF: with the use of the cutoff value of 8,296 ng/L, the sensitivity of HF diagnosis was only 52% while the specificity was 79% (see Figure 2 for more details). The sensitivity and specificity increased only slightly when only symptomatic HF patients were included (NYHA 2–4) (Figure 2).

**Table 5:** Relation of NTproBNP to other variables.

	*R*-value	*p*-Value
Age	0.21	0.003
Dialysis vintage	0.17	0.02
Residual diuresis	−0.21	0.009
Left ventricular ejection fraction	−0.22	0.03
Left ventricular mass index	0.28	0.0001
Left atrial volume index	0.56	<10^−5^
Tricuspid annular plane systolic excursion	−0.12	0,12
Right atrial end-diastolic volume	0.45	0.0003
Estimated pulmonary artery systolic pressure	0.50	<10^−5^
Central venous pressure	0.28	0.0002
AVF flow volume	−0.03	0.97
Cardiac index	−0.18	0.02
Effective cardiac index	−0.20	0.009
Systemic vascular resistance	0.24	0.004
Total vascular resistance	0.22	0.008
Mean arterial pressure	0.04	0.56

Note that the NTproBNP values were related especially to atrial volumes and left ventricular mass.

## 4. Discussion

The main findings of this study are as follows: (1) HF was highly prevalent in ESKD patients on chronic hemodialysis. HFpEF and HOHF were the leading phenotypes. (2) HF and especially HFpEF were underdiagnosed by nephrologists. (3) Normal left ventricular geometry was found in only 20% of patients, which explained the high prevalence of diastolic dysfunction. (4) NTproBNP was not a precise marker of HF in patients on chronic hemodialysis.

Our study diagnosed HF of any phenotype in 57% of all patients on maintenance hemodialysis. For comparison, Harnett et al. (17) reported a 31% prevalence of HF among hemodialysis patients in a study that started in 1982 (i.e., before the concept of HFpEF) and included significantly younger patients. In 2004, Cheung et al. (18) reported HF in 40% of prevalent hemodialysis patients in the HEMO study, again on younger patients. Stack and Bloembergen (19) found HF in 36% of patients entering chronic hemodialysis, while Antlanger et al. (20) reported recently a 70% prevalence of HF. Although we could explain the differences by including older and more complicated patients in the hemodialysis programs nowadays, the main difference is most probably in the definition of HF itself: Harnett et al. (17) used a clinical HF diagnosis (without the need for echocardiography), and in the Stack’s study (19), patients were recorded as having HF if the diagnosis occurred in the discharge letters. The more recent study by Antlanger et al. (20) used the 2012 ESC guidelines for HF diagnosis (21). Moreover, the older studies did not specifically rule out overload that could mimic HF. Nevertheless, the diagnosis of HF was associated with increased mortality (17, 19, 20).

Metabolic syndrome is a known risk factor of HFpEF in the general population (22). In this study, two main etiologies of CKD are included, i.e., type 2 diabetes mellitus and hypertension, which are parts of the metabolic syndrome. Moreover, the high very prevalence of HFpEF was also not surprising in light of the very frequent left ventricular hypertrophy (LVH) or concentric remodeling and diastolic dysfunction in this study. Similar findings were also reported by Antlanger et al. (20), although they used HFpEF diagnosis differently from the recent one. LVH is linked to increased mortality (23), and many causes of LVH were identified in ESKD patients, such as arterial hypertension and cyclic fluid overload (24), and also higher levels of the fibroblast-growth factor-23 (25). Indeed, the histological structure of LVH in ESKD patients differs from LVH of other etiologies (26). Nowadays, echocardiography could detect myocardial fibrosis of both ventricles and of the left atrium through speckle-tracking (strain analysis) (27). Interestingly, HFpEF (and HOHF) were the most frequent phenotypes underdiagnosed by the referring nephrologists. It highlights lower awareness of these two phenotypes by non-cardiologists that are known also in the non-CKD population (28). HOHF is a specific HF phenotype that was even missed by the guidelines (29). A total of 60% of our HOHF cases had high dialysis fistula flow (>1500 ml/min), which was considered the main reason for HOHF. They were indicated for flow-reducing surgery. Other known contributing factors in developed countries include obesity, anemia, or hepatic disease. Also, HOHF is linked to higher mortality (30). High-flow AVFs have recently been associated with increased myocardial fibrosis (31).

NTproBNP levels increase with the worsening of glomerular filtration in CKD patients (32), and, also in our study, the levels were very high. Although the NTproBNP levels were higher in HF patients and in patients with respective HF phenotypes than those in non-HF controls, NTproBNP is not a good HF marker according to our study due to high false-positive rates. Claus et al. (33) did a similar conclusion, and according to their findings, the sensitivity and specificity could increase by adding other biomarkers, such as growth differentiation factor-15 and circulating neprilysin. Interestingly, NTproBNP values were related especially not only to the volumes of both atria and left ventricular mass index (see Table 5), i.e., to variables that mirror long-term volume overload, but also to higher AVF flow (34). A similar conclusion was published recently (35). Although it is not sure whether NTproBNP level is a marker of HF in ESKD patients or not, a recent meta-analysis documented a gradual increase in cardiovascular and all-cause mortality in ESKD patients with high NTproBNP values (36). Moreover, increased neprilysin levels independently predicted the composite of CV events and cardiac events in HD patients (37).

The main limitation of this study is that the hemodynamic data were obtained non-invasively, but the wide use of the right heart catheterization is no more justifiable nowadays. Although we made every effort to examine only patients with optimal hydration, improper dry weight setting could have played a role in some cases. Moreover, this study was performed in one European country, and 98.5% of the included patients had Caucasian race. The next limitation is the cross-sectional design of this study and inclusion of prevalent patients with various time spent on hemodialysis.

## 5. Conclusions

Heart failure is present in more than half of ESKD patients on chronic hemodialysis, but it was frequently missed by the nephrologists. HFpEF and HOHF were the leading phenotypes. Impaired left ventricular geometry occurred in the vast majority of patients, and it probably explained the high prevalence of HFpEF. Based on the current knowledge, adequate dry weight setting and avoidance of high-flow AVFs could be causal precautions, especially in patients with HFpEF. NTproBNP is not helpful in HF diagnosis among patients on chronic hemodialysis.

## Data Availability

The raw data supporting the conclusions of this article will be made available by the authors, without undue reservation.
